# Digitalizing breeding in plants: A new trend of next-generation breeding based on genomic prediction

**DOI:** 10.3389/fpls.2023.1092584

**Published:** 2023-01-19

**Authors:** Donghyun Jeon, Yuna Kang, Solji Lee, Sehyun Choi, Yeonjun Sung, Tae-Ho Lee, Changsoo Kim

**Affiliations:** ^1^ Plant Computational Genomics Laboratory, Department of Science in Smart Agriculture Systems, Chungnam National University, Daejeon, Republic of Korea; ^2^ Plant Computational Genomics Laboratory, Department of Crop Science, Chungnam National University, Daejeon, Republic of Korea; ^3^ Genomics Division, National Institute of Agricultural Sciences, Jeonju, Republic of Korea

**Keywords:** QTLs, GWAS, MAS, genomic prediction, machine learning, deep learning, high throughput phenotyping, AI breeding

## Abstract

As the world’s population grows and food needs diversification, the demand for cereals and horticultural crops with beneficial traits increases. In order to meet a variety of demands, suitable cultivars and innovative breeding methods need to be developed. Breeding methods have changed over time following the advance of genetics. With the advent of new sequencing technology in the early 21st century, predictive breeding, such as genomic selection (GS), emerged when large-scale genomic information became available. GS shows good predictive ability for the selection of individuals with traits of interest even for quantitative traits by using various types of the whole genome-scanning markers, breaking away from the limitations of marker-assisted selection (MAS). In the current review, we briefly describe the history of breeding techniques, each breeding method, various statistical models applied to GS and methods to increase the GS efficiency. Consequently, we intend to propose and define the term digital breeding through this review article. Digital breeding is to develop a predictive breeding methods such as GS at a higher level, aiming to minimize human intervention by automatically proceeding breeding design, propagating breeding populations, and to make selections in consideration of various environments, climates, and topography during the breeding process. We also classified the phases of digital breeding based on the technologies and methods applied to each phase. This review paper will provide an understanding and a direction for the final evolution of plant breeding in the future.

## 1 Introduction

By 2050, the world’s population is expected to reach 9.6 billion, which requires large agricultural production. Consequently, it will be necessary to increase agricultural production by more than 70% (Godfray, 2014). In addition to direct causes, such as population growth, the indirect causes include the appearance of rapidly developing countries leading to urbanization and modernization and their population’s diet shift towards dairy and meat products. The increased demand for animal-based diets promotes higher cereal crop consumption than a vegetarian diet due to feeding cereal crops to animals. Therefore, without changes in crop production, unequal food distribution among the world’s population will deepen (Bradshaw, 2017). Moreover, the world’s climate is changing rapidly due to global warming. The average temperature of the world rises every year and abnormal climate phenomena occur (Zandalinas et al., 2021).. High temperatures and unpredictable precipitation patterns create challenges for crop growth. Therefore, a strategy to increase agricultural production in preparation for climate change is essential. Considerable progress has been achieved in producing crops tolerant to biotic and abiotic stress. Moreover, several attempts to increase the economically beneficial traits of crops in the 20th century were successful (Crossa et al., 2017). However, despite these improvements, the recent increase in yields of major crops is not enough to meet the expectations of future agricultural production demands. In order to increase agricultural production, it is essential to develop new cultivars that are resilient to climate change and have increased yields. Moreover, economic growth in many countries increased the demand for horticultural crops. As a result, horticultural crops, particularly fruits and vegetables, which are indispensable to us, increase our interest in improving our health and quality of life. Therefore, agricultural researchers worldwide must use various breeding methods to improve varieties, increase production of all crops and horticultural crops, and develop new breeding techniques and varieties using new technologies to meet consumers’ requirements.

In the second half of the 20th century, a backcrossing breeding method was commercialized. Marker-assisted backcrossing (MABC) allows rapid introgression of key genes representing superior traits into elite cultivars or breeding lines, resulting in a cultivar containing both the transgene and the preferred alleles (Ragot et al., 1995). MAS genetically enhances useful traits in crops that are difficult to phenotype. In addition, the fixation of the transgene in commercial cultivars can proceed rapidly (Moose and Mumm, 2008). However, MAS is effective for a few quantitative trait loci (QTLs) with a significant effect on the trait but not for traits dominated by numerous QTLs with minor effects. Therefore, researchers try to solve these constraints. As a result, GS emerged as an alternative. GS estimates the value of breeding based on markers distributed throughout the genome and does not use just a few markers like traditional MAS (Meuwissen et al., 2001). While MAS completely depends on molecular markers associated with target traits, GS resembles conventional breeding methods that depend on phenotypes and the breeder’s selection ability. In conventional breeding, breeders select plant individuals based on their preferences and experience. On the contrary, MAS objectively selects plant individuals with molecular markers. In the GS procedures, the genetic or breeding population is mechanically trained based on statistical models (training population). Then, the model is applied to the breeding population for selection (breeding population or validation population). Provided that training is the process of breeders gaining experience, the application of the model may be the process of breeder’s selection. Consequently, GS can be more reasonable for breeding complex traits to which a number of minor genes are related.

Humans have bred crops to introgress useful traits and increase yields. Breeding methods have changed from domestication to traditional phenotypic selection, molecular breeding, and phenotype prediction (Razzaq et al., 2021). The recently proposed GS in plants is more difficult to predict the effect of environmental variables than animals and most of the processes are still labor-intensive. So, it is difficult to apply GS to actual breeding selection yet. In order to solve this problem, several studies have conducted GS using ML technology. Some agricultural scientists call these attempts digital breeding to distinguish them from previous GS. However, digital breeding is still in its infancy and lacks a precise definition, causing much confusion in communication among researchers. In order to solve these controversies, we would like to define a new term digital breeding. Digital breeding means that breeding design, experimental plot arrangement, growth process, and selection are all carried out automatically in the breeding process while predicting and considering various environmental variables. Digital breeding aims to automate the breeding process of plants by minimizing human intervention. Recently, biological data has been digitized and increased. In the same way, breeding technologies adopt some cutting-edge sciences such as next-generation sequencing, machine/deep learning, speed breeding, and advanced statistical models, enabling predictive breeding. This article surveys breeding technologies to date, classifies the concept, application, and research status of digital breeding, and attempts to provide clarification by borrowing the idea of the levels of driving automation. In this review article, the past, present, and future of breeding is divided into six stages according to the level of technological development. It will be helpful to understand breeding trends so far and to present the direction and achievements of breeding in the future.

## 2 Outline of breeding techniques

### 2.1 Traditional breeding techniques

Plant breeding developed through efforts to establish better-performing varieties by crossing between cultivars with the traits desired by the breeder (Acquaah, 2009). Breeders select phenotypes with desired traits, such as semi-dwarf and nutrient-efficient plants. Plant breeding aims to select, identify, and expand varieties with valuable traits desired by consumers and breeders in the next generations by targeting diversity through new variations. Therefore, breeding will fail if suitable individuals cannot be selected for the next generation. So for breeding success, breeders studied various breeding methods, considering the multiple characteristics: fertilization method, breeding ability, combination ability, breeding scale, and breeding age. In addition, the breeding method was determined through the genetic structure of the cultivars’ traits and the degree of interaction between genes (Kearsey, 1997). Breeding methods considering these factors can be classified into segregation breeding and cross-breeding (**Table 1**). Segregation breeding nurtures excellent individuals or groups while selecting outstanding individuals or lines when a genetically diverse group has already been secured. The pure line selection method and the mass selection method are representative. Cross-breeding is breeding cultivars with an excellent performance by recombining or introducing beneficial or desired genes through an artificial cross between cultivars or lines. Pedigree selection, bulk population, single-seed descent (SSD), and backcross breeding are representative methods in cross-breeding.

**Table 1:** Comparison of breeding methods according to pollination of plants.

Methods	Self-pollinated	Cross-pollinated
Pure line selection	O	
Mass selection	O	O
Pedigree selection method	O	O
Bulk population method	O	O
SSD	O	
Backcross breeding	O	O

#### 2.1.1 Pure line selection

In 1903, Johannsen’s research confirmed that a population mixed with self-pollinated species could be classified as a genetically pure line. Pure lines are suitable for applications that require trait uniformity because of small genetic differences and similar phenotypes (Acquaah, 2009). Furthermore, pure line selection is rapid, and genotype selection from a diverse population can continue to repeat selfing until there is no apparent segregation in subsequent generations (Poehlman and Sleper, 1995). Based on these results, selection can eliminate variation but not create variations. Thus, the variations produced in the pure line are caused by environmental factors, meaning that selection in the pure line is meaningless. In addition, pure line cultivars are virtually challenging to produce in diverse environments due to their small genetic differences. Therefore, pure lines play an important role as a material for cross-breeding or generating genetic populations.

#### 2.1.2 Mass selection

Mass selection applies to self-pollination and cross-pollination, but the genetic results differ (Allard, 1960). The continuation of inbreeding changes the gene frequency of the population. It decreases the heterozygosity from one generation to the next. Still, there is little change in allele frequency in cross-pollination unless the allele associated with the desired trait is changed through selection (Acquaah, 2009). This method is a population improvement strategy aiming to increase the average performance of the base population by increasing the gene frequency of the desired gene and acting on existing variabilities without creating new ones (Acquaah, 2015). The general process of mass selection can be divided into two categories. One is the negative mass selection to remove plants with unwanted traits or off-types, and the other is the positive mass selection to select plants with desirable traits to maintain purity and to generate more plants with desired traits (Acquaah, 2009). These processes can be performed based entirely on the breeder’s visual judgment, either directly by selecting the desired trait or indirectly by selecting traits related to the desired trait (Gjedrem and Thodesen, 2005). Also, since the selection is based on the plant’s phenotype, it is preferable if the desired trait is highly heritable or controlled by additive genes (Brown and Caligari, 2011). Although the resulting plants are relatively homogeneous, they are genotypically broad, containing different pure lines. Since only one generation is required per cycle, this method is inexpensive, simple, and fast.

#### 2.1.3 Pedigree selection

Pure line selection and mass selection methods focus on genetic variation, whereas pedigree selection methods create variation through hybridization. A pedigree selection method can be used for cross-pollinating species, but it is mainly used for breeding self-pollinating species. Pedigree selection uses a pedigree with accurate records of breeders to keep the ancestors by knowing who the parents of the F2 generation are in order that F2 individuals can be identified through the descendants of subsequent generations (Poehlman and Sleper, 1995). As a result, a base population is established through parental hybridization, followed by selection and isolation, which progresses through generations until a desired degree of homozygosity is achieved (Allard, 1960). Breeders can also influence genetic diversity and variation through these selections and processes. In this method, selecting species that are easy to observe, select, and harvest is desirable. Since lines begin to form after F4, selection should be based on progenies rather than individuals.

#### 2.1.4 Bulk population

The bulk population method improves crops by putting off artificial selection until later generations and influencing variation through natural selection at the early generation stage (Briggs and Knowles). In other words, it is a breeding method in which homozygosity of the population is increased by repeating mixed breeding and group cultivation without selection in the early generation of hybrids (F2 to F4) and then fixing the line through individual selection in later generations (F4 to F5) (Allard, 1960). The rationale for this method is that natural selection will eliminate individuals sensitive to various abiotic stresses in the production area for which traits are not desirable (Acquaah, 2015). Varieties produced by this method will have already adapted to the place of production, and the method can be applied to creating cross-pollinated inbreeding populations. Still, breeding self-pollinating species that grow in tight spaces between individuals is best. However, the approach is that the genotype of the desired trait can be lost in early generations if it is not competitive under natural selection. In contrast, the genotype of a competitive but unwanted trait under natural selection can persist into future generations (Acquaah, 2009).

#### 2.1.5 SSD

SSD is a compromised breeding method between the bulk population and pedigree selection. This breeding method randomly selects ‘only one seed’ from each plant of the F2 population with the desired trait created by artificial hybridization of F1 to shorten the breeding time and reduce genotype loss in the segregated generation (Allard, 1960). This advances the generation of many F2 plants through multiple generations, enabling rapid cultivation and selection of lines with high homozygosity in later generations. This method is best for plants such as soybeans that can self-pollinate and be grown densely (Tigchelaar and Casali, 1976) and can be used in small spaces such as greenhouses. However, it is not suitable for traits with low heritability, polygenes, or traits associated with pleiotropic genes.

#### 2.1.6 Backcross breeding

Backcross breeding aims to transfer one or more specific genes of interest from an ineffective breed to an excellent breeding line while preserving all other characteristics in the already excellent breeding line. It is a breeding method achieved over a relatively short time through repeated crosses and selection of superior breeding lines, i.e., recurrent parents (Acquaah, 2009). Although this method is best suited for qualitative traits (Borojevic, 1990), backcrossing may be difficult if the gene of interest is closely associated with unwanted genes, resulting in linkage drag (Allard, 1960).

### 2.2 Techniques in molecular breeding

**Figure 1:**
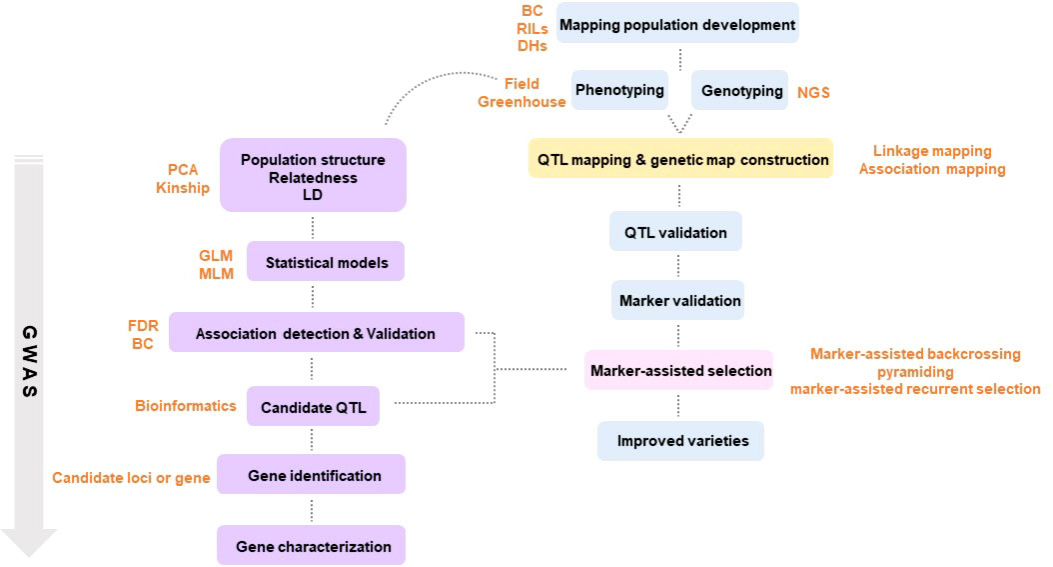
Schematic diagram of plant breeding using molecular markers.

Traditional breeding, with a history of nearly 100 years, liberated humanity from starvation with groundbreaking achievements. Despite these outstanding achievements, it stagnated due to a long breeding time and the limited number of crops that could be cross-bred. Amid these difficulties, a new concept emerged that applied a molecular-based selection strategy that replaced the existing phenotype-based breeding strategy. Until then, breeders visually made selections for breeding. However, after the advent of molecular breeding, it became possible to determine whether the genes of the parent generation were passed on to the next generation through gene analysis technology (Savadi et al., 2018). Next-generation sequencing (NGS) enables the discovery of molecular markers by sequencing the entire genome of a plant. Molecular breeding makes it possible to accurately select useful individuals with desired traits without being greatly affected by the environment by using molecular markers that detect the traits of each individual (Metzker, 2010). Modern molecular breeding is a powerful molecular tool based on precision breeding that can reduce the time and effort required to produce new varieties and provide new and accurate in-depth research to satisfy the increasing global population and large-scale plant breeding requirements (**Figure 1**).

#### 2.2.1 QTL mapping

Molecular markers with DNA-based polymorphisms can be used for genetic improvement by allowing for the selection of useful traits (Tewodros, 2016). In general, most of the agriculturally important traits are quantitatively inherited. Their genetic variation can be attributed to the collective response of several small effects associated with the trait. Methods utilizing traditional molecular markers include finding QTL-associated markers that regulate the expression of any trait in single or multiple parental mapping populations (Semagn et al., 2015). Throughout the 1980s and 1990s, various rich molecular markers were developed, enabling QTL mapping with reasonable marker density and genome coverage (Cooper et al., 2004). In plants, QTL mapping enriches biological knowledge of genetics and genetic structures across related species, providing useful markers to understand the genetic structures of complex traits (Bernardo, 2008). Therefore, building linkage maps and finding correlations between genetic markers and phenotypic traits is fundamental (Wang et al., 2016). Relatively simple single-marker analysis and more sophisticated interval mapping (Haley and Knott, 1992), joint mapping (Kearsey and Hyne, 1994), multiple regression (Whittaker et al., 1996) and composite interval mapping (Zeng, 1994) are used in various ways to link genotypes with many different quantitative traits. In recent decades, QTL mapping studies identified various QTLs that regulate complex phenotypic traits in crops such as rice (Yano et al., 2000), maize (Yano et al., 2000), arabidopsis (El-Assal et al., 2001), and tomato (Fridman et al., 2000).

#### 2.2.2 Linkage disequilibrium (LD) mapping

Another method of selection to identify loci involved in the inheritance of complex traits is association mapping, also called LD mapping. This methodology is more efficient than QTL mapping, as it explores diversity using existing natural populations or germplasm collections with diverse cultivars, as opposed to QTL analysis, which uses bi-parental populations constructed using contrasting parents (Gómez, 2011). Correlation between mapped genetic markers and traits can be used to detect QTLs (Ibrahim et al., 2020). This can be difficult to do unless you have a well-annotated genome, as it allows for detecting more alleles with high resolution and precise mapping of quantitative traits but requires extensive knowledge of markers within the genome.

#### 2.2.3 Genome-wide association study (GWAS)

With the development of NGS technology, computational methods using information from the genetic analysis have been improved, and GWAS search for key genes underlying important traits, contributing to the production of genetically improved plants. The first published GWAS was a study on humans in the early 2000s. Since then, several active studies in animals and plants have been conducted (Ozaki et al., 2002). This methodology has emerged as an alternative approach to bi-parental QTL mapping in various crop species. Research on associations between molecular markers (e.g., SNPs) and desired phenotypic traits is key to identifying relevant genes. In addition, there is no need to develop new mapping populations because historical recombination events between accessions are used to find relevant genomic regions (Nakano, 2020). Once accessions are genotyped, the data can be used for many different traits, enabling quick research of different traits in various environments (Deng et al., 2021). In the case of GWAS, since the genetic variation is identified by genotyping many markers, it is important to select an optimal statistical model to detect false positives. Consequently, statistical power and computational efficiency may be indispensable for detecting truly associated markers (Wu and Zhao, 2009). In GWAS, the mixed linear model (MLM) and general linear model (GLM) are the most recommended. In addition to those, there are various models such as the compressed MLM (CMLM), enriched CMLM (ECMLM), and the settlement of MLM under progressively exclusive relationship (SUPER), which are single locus analyses similar to GLM and MLM but more advanced. There are also the multiple loci mixed linear model (MLMM) and the fixed and random model circulating probability unification (FarmCPU). Among them, GLM is one of the methods with high computational efficiency, which can lower false positives by using the population structure and principal components as fixed effects. However, if the polygenic background is not sufficiently calculated, the false positives will be high, and the family structure will not be considered in the statistical analysis (Mebratie et al., 2019). However, MLM (kinship or kinship + Q matrix + PCA) takes into account the population structure and is used to control for false positives by virtue of familial relatedness. These MLMs perform better than the GLM model alone and are widely used as an alternative to GLM (Alqudah et al., 2020). These models were improved and developed to produce better statistical results. However, MLM can be difficult to control for false positives when it involves population structures with extensive genetic diversity. In this case, CMLM and ECMLM were developed to increase statistical power further. The SUPER model increases statistical power by inducing kinship using relevant genetic markers instead of the entire markers. MLMM and FarmCPU extend the single-loci method of MLM to a multiple-loci method. FarmCPU combines the MLMM strategy into the limited kinship matrix of the SUPER model (Kusmec, 2018). Thus, it tests markers using multiple related markers as covariates in a fixed effects model and performs analysis on related covariate markers in a random effects model. FarmCPU is faster than MLMM and effectively improves problems caused by false positives (Liu et al., 2016). In addition, as new models are continuously developed, the performance and statistical power of GWAS is expected to increase over time. Also, existing models need to be improved more efficiently in line with increasing data availability.

#### 2.2.4 MAS

Recently, the amount of molecular markers for traits of interest in plant breeding has been gradually increasing. QTL mapping to identify genetic loci quantitatively associated with traits of interest is the basis for developing molecular markers used in MA) (Ibrahim et al., 2020). MAS can be defined as the manipulation of a genomic region involved in expressing a trait of interest in a short time through the application of DNA markers. These attempts led the study of molecular breeding into a new era (Sharma, 2020). It is also applied to plant breeding to improve tolerance to biotic or abiotic stress and to improve crop yield and quality. MAS has the basic idea of ​​using LD between markers and QTLs: using a non-random association between the marker and the QTL allele (Hospital, 2009). Identifying genes in the target trait and markers linked to QTLs is a prerequisite for these MAS (Khush, 2000). The framework of MAS in plant breeding is divided into four groups: (i) marker-assisted backcrossing, (ii) marker-assisted pyramiding, (iii) early generation marker-assisted selection, and (iv) marker-based recurrent selection. These systems characterize genetic material in early segregating generations and strongly anchor the breeding cycle (Nadeem et al., 2018). Marker-assisted backcrossing (i), first described in 1992, is a technique for the introgression of one or several major genes of a donor line into the genetic background of an elite line or recurrent line. With the help of molecular markers, it was possible to speed up the selection and genomic recovery of recurrent parents (Muranty et al., 2014), and it is widely used to eliminate undesirable traits (ex., disease susceptibility) in popular varieties (Sharma, 2020). Marker-assisted pyramiding (ii) is the process of combining multiple genes into one genotype. The most common strategy of pyramiding is to combine several resistance genes to biotic and abiotic stress, which is a strategy to prevent the decay of resistance to specific diseases or stresses. This method efficiently transfers genes into improved varieties by pyramiding the gene combinations. Early generation MAS (iii) has the advantage of selecting markers at an early generation, allowing attention to fewer important lineages in the next generation compared to the previous generation (Collard and Mackill, 2008). In plants, a technique that helps to improve quantitative traits by repeating crosses and selection is called recurrent selection. The goal of marker-based recurrent selection (iv) with such recurrent selection is to augment favorable alleles and more QTLs in the population before inbred lines extraction (Bankole et al., 2017). This method can efficiently breed complex traits because it can use minor genes/QTLs that do not significantly affect the phenotype (Abdulmalik et al., 2017). However, the limitation of MAS in plant breeding is that traits composed of many minor effect alleles cannot be efficiently selected. Besides, linkage drags occur in every breeding effort, which hampers the improvement of target traits. Consequently, predictive breeding procedures using agro-bigdata and advanced statistical models embedded in some basic machine learning algorithms are being recently used for overcoming the limits of MAS, which will be discussed in the next section.

### 2.3 GS

Complex quantitative traits are regulated by genome-wide minor effect alleles. Most economically useful traits, such as yield, fruit quality, and stress tolerance, are mostly complex quantitative traits (Bhat et al., 2015). There are two major marker-based breeding methods—MAS and GS—to select plants with superior traits. In MAS, crops are selected based on QTLs detected through linkage mapping (LM) or GWAS (Myles et al., 2009). MAS cannot identify genes with minor effects associated with complex traits, and if the associated markers constitute a small fraction of genetic variation, the results are worse than phenotypic selection (Xu and Crouch, 2008). Moreover, in MAS, only a few statistically significant specific markers are used, and the rest are excluded from the analysis. Therefore, in traditional MAS, the number of specific markers per trait is generally low, and the use of MAS is limited when several genes with small effects are involved in one trait (Bernardo, 2008). Therefore, MAS is optimized for qualitative traits, such as specific metabolites and disease resistance, rather than quantitative ones.

A new method called GS deals with these problems. Improvement of complex traits requires phenotypic evaluation at various locations and multi-years to confirm the correlation between environment and genotype. However, it was difficult due to the lack of cost and labor. With the development of NGS technology, sequencing costs have become cheaper, and high-resolution genome information can be easily obtained. Advances in sequencing methods have made GS possible (Gorjanc et al., 2015). All available high-performance markers in the genome can be used to select GS crops (Jannink et al., 2010). Given a marker set covering the entire genome, GS models consider all markers influencing a trait regardless of a specific threshold. Traditional MAS focuses on a small number of major genes or QTLs, whereas GS makes predictions by integrating all available markers in the genome into the model. Calculating all genetic effects prevents the loss of genetic variance occupied by minor genes or QTLs. Therefore, GS is more effective than MAS for traits regulated by multiple markers (Meuwissen et al., 2001). The most significant advantage of GS is that it can predict the phenotype information of mature individuals based on genotype data obtained from seeds or seedlings. This process eliminates the need for comprehensive phenotypic evaluation by year or environment and increases the speed of crop varietal development (Bhat et al., 2016). Previous studies showed that compared to MAS, the accuracy of genomic prediction (GP) is three times higher for maize (*Zea mays* L.) and two times higher for wheat (*Triticum aestivum* L.) (Heffner et al., 2009). Therefore, it is expected that this genome-based predictive selection has the potential to replace phenotypic selection or marker-assisted breeding.

**Figure 2:**
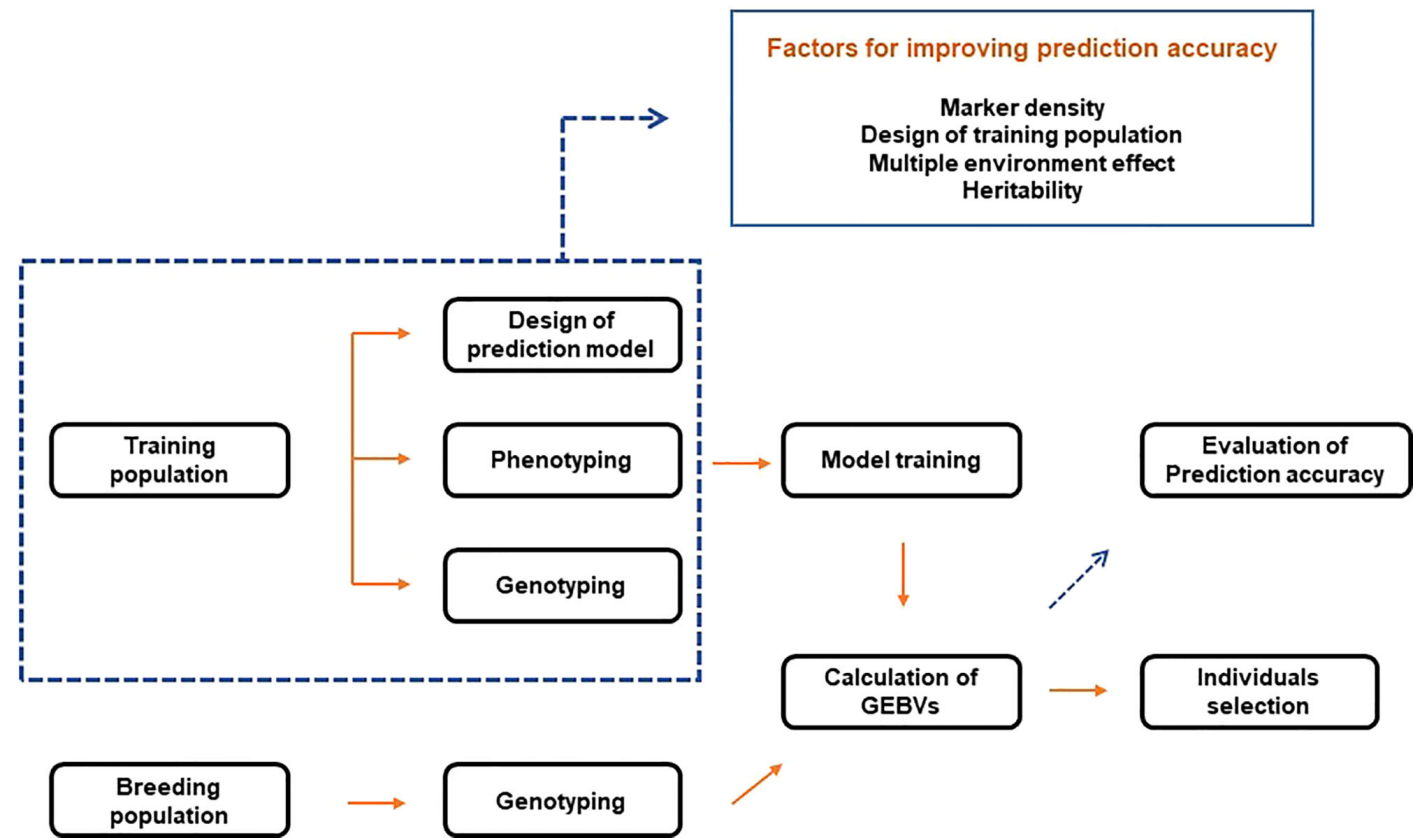
Schematic diagram of genomic selection.

GS combines phenotypic and genotype data of the training population to construct a model. Then, based on the learned model, the genomic estimated breeding values (GEBVs) of individuals in the breeding population are predicted (**Figure 2**). Therefore, with genotype data, GS selects individuals based on GEBVs from the validation (or breeding) population. In this case, phenotype information from the validation population is not required (Meuwissen et al., 2001). Breeding values consist of two elements. The first is the average breeding values of the parents, and the second is the variance of the progeny from the mean breeding values of both parents due to Mendelian sampling. Since GS uses a high density of markers to quantify Mendelian sampling, large-scale phenotypes of the progeny are not required. This process reduces the breeding cycle and is more efficient than comprehensive phenotyping. GS is effective for complex traits with low heritability and simple traits with high heritability. In addition, it is possible to reduce the development cost of a hybrid or breeding line (Crossa et al., 2017). The process of training models resembles that of acquiring breeding resources by breeders. Therefore, GS is closer to a conventional selection procedure. On the other hand, selection in the early stages of growth based on the trained models can be made, like MAS, combining the advantages of conventional breeding and MAS.

**Figure 3:**
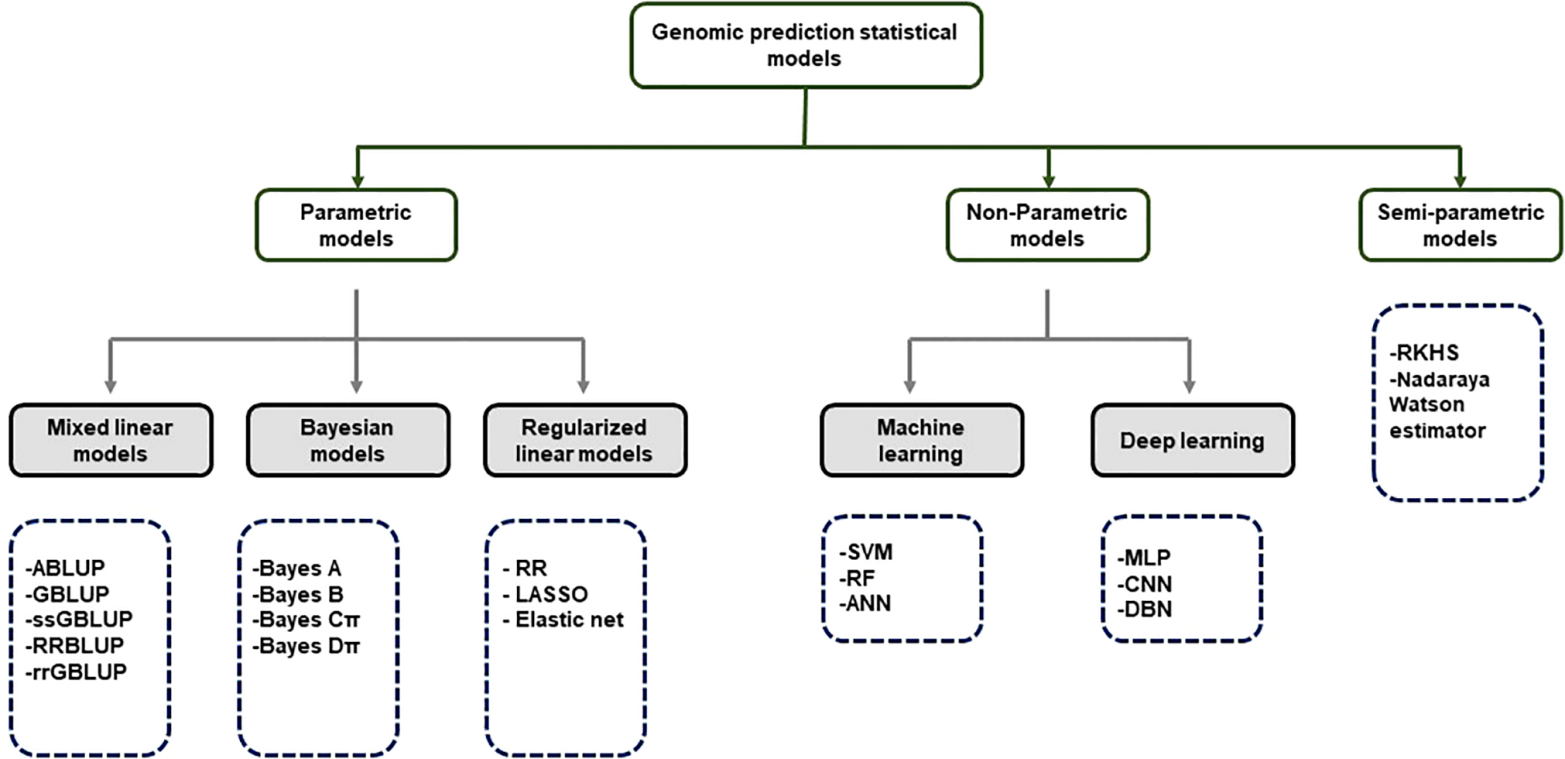
Classification of GS statistical models.

Although GS is a useful tool in plant breeding, there is limited information on setting up an optimized statistical model. Incorrect data imputation, unexpected responses, and environmental constraints limit the performance of GP. Although attempts were made to consider and overcome these limitations in several statistical prediction models, it is still the most challenging problem in multidimensional genomic data (Budhlakoti et al., 2020). The prediction accuracy difference between the respective models is determined according to the underlying statistical methods. Many GP models use a large set of markers to predict the phenotype, and each model makes different assumptions according to the distribution and difference of the markers (Goddard, 2009). So far, the only solution to avoid this limitation may be repeated trials with different statistical models to find an optimized scenario that can be applied to target traits. GP models are divided into parametric and non-parametric methods according to the presence of prior information and the setting of parameters (Budhlakoti et al., 2022). In parametric methods, Regularized Linear Regression (RLR) models such as least absolute shrinkage and selection operator (LASSO) (Tibshirani, 1996) and ridge regression (RR) (Meuwissen et al., 2001) emerged, improving the over-parameterization problem of the existing simple linear model. Currently, best linear unbiased prediction (BLUP) and Bayesian models are mainly used according to variance assumption in GS (Meuwissen et al., 2001). Semi-parametric methods such as reproducing kernel Hilbert space (RKHS) and Nadraya-Watson estimator predict GEBVs taking into account epistatic genetic structure (Gianola et al., 2006). Furthermore, ML-based statistical models such as SVM (Long et al., 2011), artificial neural networks (ANN) (Gianola et al., 2011), and random forest (RF) (Holliday et al., 2012) have been applied to plant breeding (**Figure 3**). Various studies have tried to find optimal accuracy by applying various statistical models for each crop. However, it is difficult to define an optimal statistical method due to differences in crops, cultivars, environments, populations, and markers. Therefore, it is necessary for breeders to compare and to select appropriate statistical methods for each situation when conducting GS.

ABLUP- Traditional pedigree- Best Linear Unbiased Predictor; GBLUP-Genomic Best Linear Unbiased Predictor; ssGBLUP-Single-step Genomic Best Linear Unbiased Predictor; RRBLUP-Ridge Regression Best Linear Unbiased Predictor; rrGBLUP- Ridge Regression Best Linear Unbiased Predictor; Bayes-Bayesian method RF-Random Forest; RR-Ridge Regression; LASSO-Least Absolute Shrinkage and Selection Operator; SVM- Support Vector Machine; RF-Random Forest; ANN-Artificial Neural Networks; MLP-Multi-Layer Perception; CNN-Convolutional Neural Networks; DBN-Deep Belief Network; RKHS- Reproducing Kernerl Hillbert Space.

#### 2.3.1 RLR models

GS tries to avoid biased marker prediction effects by using the entire marker. Ordinary least squares (OLS) is a simple method for measuring the influence of markers. However, OLS only makes predictions within a sample and does not allow the weighting of markers. In the case of a high density of markers, the number of markers (p) exceeds the sample size (n). Therefore, it is not appropriate to obtain an estimate of the marker effect through OLS (Perez et al., 2010). In order to solve this problem, RLR models were proposed, such as RR and LASSO. The RLR model reduces the variance of regression coefficients by shrinking the regression coefficients. Through this process, only the key predictor variables are considered when making estimations in the statistical model (Szymczak et al., 2009). This process can improve prediction accuracy by reducing the mean squared error (Friedman et al., 2010).

RR estimates the regression coefficient using parameter shrinkage, giving the ℓ2-norm penalty. The ℓ2 penalty of RR tends to shrink the coefficient to zero. In particular, RR is advantageous for non-zero coefficients. In the case of k identical predictor variables, when the predictor variables are fitted alone, each coefficient is equally reduced to the size of 1/k. Moreover, RR is effective when many predictors have small effects, especially coefficients with many correlated variables. Therefore, RR cannot eliminate coefficients and cannot select only a relevant subset of predictors. Similar to RR, LASSO is advantageous for processing large amounts of data quickly and efficiently (Friedman et al., 2010). However, LASSO has a weakness when many predictors are correlated. If only one suitable predictor is selected from k identical predictor variables, the others are ignored. That is, Lasso’s ℓ1 penalized least squares criterion excludes coefficients close to zero. The most significant difference between LASSO and RR is that ridge regression only shrinks coefficients, whereas LASSO also selects variables. In addition, LASSO shrinks coefficients with the same force for all coefficients, but RR shrinks coefficients in proportion to the size of the coefficients (Ogutu, 2012). On the other hand, elastic net combines the penalties of LASSO and RR. Elastic net penalty weights LASSO and RR. When the penalty parameter is close to 0, it corresponds to RR. However, when it is close to 1, it shows a performance similar to LASSO. Elastic net solves the disadvantages of using RR or LASSO alone and shows high prediction accuracy when predictors are correlated (Tutz, 2009).

#### 2.3.2 MLM

Estimating the value of breeding requires optimizing the estimation of the regression coefficients but also optimizing the information in the phenotypic data. There is a method that combines these two factors. There is a method of obtaining a phylogenetic effect by simultaneously modifying the phenotype and a method of estimating the breeding value using an additional genetic relationship between plants. This method is called best linear unbiased prediction (BLUP) (Goldberger, 1962). BLUP statistical method was proposed by Henderson (1975) as the coefficient matrix of mixed model equations (MME) for linear mixed models used in data analysis to estimate and predict random effects. BLUP evaluates all objects simultaneously and acts as a linear model, correcting for different effects between objects and estimating by combining objects and fixed effects. For linear models, a single genotype effect appears as an independent random variable, uncorrelated (Piepho et al., 2008). These BLUP models include pedigree-based BLUP (ABLUP), genomic best linear unbiased predictor (GBLUP), and ridge regression BLUP (rrBLUP). ABLUP is a standard method for predicting breeding values ​​through inferable associations between individuals using relevant information based on pedigree (Crossa et al., 2010). ABLUP’s Ep is assumed to follow a normal distribution of random additive effects (Rezende et al., 2014). GBLUP is derived from ABLUP but differs in that the matrix of the marker-based matrix Eq is replaced by G (Myburg et al., 2007). GBLUP has potential advantages over ABLUP. First, the prediction of similarity computed based on pedigree is replaced by similarity feasible in GBLUP, which consists of weak assumptions. Possible similarities can also be characterized in pairs instead of based on family history in ABLUP (de los Campos et al., 2009). Unlike the previous two methods, rrBLUP changes the parameter notation in Eq. It has a similar computational method to GBLUP and assumes that the same marker effects are reduced, and the variances are equally normal. The GBLUP method is the most common approach in animals and plants. GBLUP was parameterized with the ridge regression model (Hoerl and Kennard, 1970), rrBLUP, to predict the genome. The reduction of SNPs can be determined either by normalizing rrBLUP or by the ratio of variance components in GBLUP (Heslot et al., 2012).

#### 2.3.3 Bayesian prediction models

Bayesian analysis is named after the British mathematician Thomas Bayes. This analysis is a statistical method that combines the information in the sample with the information on the parameters in the population in advance to explain the statistical reasoning process (Gelman et al., 1995). The first step in using the model is specifying the probability distribution of the parameter of interest in advance and applying it by providing the posterior probability distribution for the parameter. These posterior distributions provide the basis for the parameters (Mila and Ngugi, 2011). In this method, single nucleotide polymorphisms (SNPs) encompass the entire genome and are used in animals and plants to estimate breeding values. Training to estimate the effect of SNPs in statistical problems allows us to estimate the effect in situations where the number of individuals is much smaller than the vast amount of SNPs (Habier et al., 2011). A key part of this analysis is the probability distribution of a parameter in the population. The Bayesian approach allows for subjective and objective data to pre-determine the distribution. Therefore, some argue that Bayesian analysis lacks objectivity (Habier et al., 2011).

Many Bayesian methods for GS have been developed. Similar sampling models have been shared, and a new concept of the Bayesian alphabet has emerged, including Bayes A and Bayes B in different analytical methods (Gianola et al., 2009). In animal and plant breeding studies, hierarchical Bayesian models such as Bayes A and Bayes B were presented (Meuwissen et al., 2001), as well as Bayesian LASSO (Legarra et al., 2011; Li and Sillanpää, 2012) and Bayesian ridge regression (BRR) (De Los Campos et al., 2013).

Direct approaches such as GBLUP first construct the relationship between SNPs and molecules and then utilize mixed model equations to determine genetic merit directly [89]. The Bayesian approach is more effective than other QTLs and genetic value prediction models, as many SNPs are preferentially merged into ineffective ones (Meuwissen et al., 2001). In addition, the Bayesian method, which assigns higher variances to subsets of SNP effects, has been shown to achieve higher prediction accuracy than GBLUP when large effect variations contribute to complex traits (Hayes et al., 2010).

Although the Bayesian model is widely used in animal and plant breeding, it has several drawbacks, including those previously described. First, SNPs treated as close to one are assigned an ineffective ratio. Second, the degree of freedom of the independent data dictionary is used for distribution. Only one degree of freedom can be added regardless of subsequent phenotypes and genotypes. In order to overcome these shortcomings, Bayes Cπ and Bayes Dπ were developed and sampled by replacing parameter π or scale parameter S with variables rather than data information (Habier et al., 2011).

Bayesian methods in this context have mechanisms to combine prior probability distributions with sample data information for later probability distributions in the natural state. Because of this, it can also be used to make better decisions in posterior probabilities.

#### 2.3.4 Semi-parametric prediction models

Parametric models such as MLM and Bayesian models, widely used in GS, use a prior effect size distribution determined by several parameters. The parameters used in predictive models limit the amount of information the model can use. Therefore, the small number of parameters determined in the predictive model limits the flexibility of the model. On the other hand, in the semi-parametric model, there is no assumption that data follows a specific distribution, and the number of parameters is determined according to the amount of training data. The semi-parametric model can be used when there is no prior information about the data (Murphy, 2012). Predictive models can be applied differently depending on the genetic architecture of the trait. Parametric predictive models can take into account additive effects but are ineffective for epistasis due to the difficulty of predicting high interactions (Howard et al., 2014). Semi-parametric and non-parametric models have high accuracy in genetic architectures with epistasis effects.

Conversely, semi-parametric models involve epistasis effects and have been used in several plant prediction studies. Epistasis plays a vital role in explaining the occurrence of genetic variation, and considering epistasis in predictive models can obtain good predictive accuracy of plant breeding in quantitative traits (Cordell, 2002; Howard et al., 2014). Epistasis is the interaction between genes in which one locus affects the phenotype by altering the effects of another locus. Therefore, epistasis can occur between multiple loci, and various interactions must be included in the model to calculate GEBVs for GS. A large number of markers is used in GS, and the corresponding epistasis interactions increase even more. Therefore, it is difficult to predict genetic gain using a model using only a small number of specific parameters (Moore and Williams, 2009).

For this reason, several models have been devised for genetic prediction. RKHS is designed for genetic prediction in non-linear models. It makes inferences about functions without prior information in a semi-parametric method. RKHS reflects independent variables in a finite-dimensional space into infinite-dimensional Hilbert spaces. This method assumes that distances in Euclidean space can be expressed through a kernel matrix that reflects the distances between objects in Hilbert space (Rodriguez-Ramilo et al., 2014). RKHS obtains prediction results by applying ML after transforming the independent variable using a kernel function. RKHS using implicit transformations has good results for predicting non-linear patterns of data (Gianola and van Kaam, 2008).

## 3 Digitalizing plant breeding

### 3.1 Strategies to increase prediction accuracy in GS

Comparison of GS methods is evaluated by prediction accuracy. The prediction accuracy is measured by the correlation between the measured GEBVs and the actually measured phenotype data. Therefore, improvement of prediction accuracy is important for GS applications.

#### 3.1.1 Marker density and selection

Among them is the density of the marker. Since GS estimates the effects of markers using LD between quantitative loci and markers, a high density of markers is advantageous for GS (Meuwissen et al., 2001). Even for low-density markers in GS, some efficiency can be guaranteed if the intervals of the markers are uniformly distributed (Habier et al., 2009; Spindel et al., 2015). In addition, markers are selected by additive effect sizes, and when the trait of interest is oligogenic, it has high predictability compared to markers with uniform spacing (Li et al., 2018). In GS, a method using high-density SNPs is widely used to increase accuracy. However, this method has a negative side when species have low importability or large populations (Crossa et al., 2010). Therefore, reducing marker density could be a solution to reducing the cost of GS implementation.

To take advantage of GS while lowering the marker density, highly correlated duplicate markers can be removed from the LD block. This approach reduces multicollinearity and does not interfere with the marker effect. It can also be a good alternative because it reduces the possibility of overfitting (Xu, 2013). The low density of markers reduces computational analysis time and allows for genotyping of more individuals at the same cost. Even if the number of markers is reduced, it can have high prediction accuracy, so a method to identify valid markers can be a reasonable strategy for GS in the future. Therefore, breeders should consider the appropriate marker density to fit their budget and time when conducting GS (Gorjanc et al., 2015). If it is difficult to reduce the number of markers using LD, the accuracy of GP can be improved by using GWAS-related markers. The presence of irrelevant markers in the GP process can reduce prediction accuracy. GWAS predicts the effect of markers based on the entire genome and selects markers statistically linked to the target trait. Therefore, the prediction accuracy can be significantly improved if GP is performed with markers associated with the target trait (Odilbekov et al., 2019). In addition, combining GWAS and GS is convenient because it does not use additional data and uses the same existing phenotypic and genotypic information (Odilbekov et al., 2019). In short, selecting an appropriate marker for GS is an important factor for improving prediction accuracy. Breeders should consider the range of markers prior to selection.

#### 3.1.2 Design of training population

The accuracy of genetic predictions is also determined by the design of the training population. Well-established training populations are important in GS. The training population consists of breeding lines with phenotype data for target traits and genotype data. After training a predictive model with markers, the prediction of GEBVs in the test population is performed using the trained model (Akdemir and Isidro-Sanchez, 2019). As NGS technology advances, genotyping costs and time continue to decrease, but the progress in phenotyping is slow. High costs and wasted labor are difficulties and limitations in plant breeding. In general, increasing the size of the training population tends to improve prediction accuracy. However, breeders should choose an optimized training population that maximizes predictive accuracy while significantly reducing phenotypic costs (Lado et al., 2013). Traditional optimization methods use random sampling. Random sampling does not increase prediction accuracy because of under- or over-represented genetic information (Bustos-Korts et al., 2016). After comparing random sampling methods, the coefficient of determination (CD) and the prediction error variance (PEV) methods have been proposed. CD shows slightly better results than PEV because CD from random samples shows genetic diversity when selecting individuals (Rincent et al., 2012). Another method of optimizing the training population is to use the genetic information of the test population when establishing the training population. The use of genetic information in the test population leads to a significant increase in prediction accuracy by applying a genetic algorithm (Lorenz and Smith, 2015; Akdemir et al., 2015). The genetic link between the training and test populations increases GP accuracy (Wientjes et al., 2013) because when the genetic distance between individuals is close, they share a common ancestry, and there is less recombination between the marker and the QTLs. Furthermore, the two groups share polymorphic loci that produce genetic variations (Habier et al., 2010). In addition, if the genetic background between groups is shared, the interaction deviation between the QTLs and the genetic background is shared (Lorenz and Cohen, 2012). Therefore, breeders should consider training the population composition of unrelated individuals if they want to increase the accuracy of GP.

#### 3.1.3 Multiple environments and heritability

Difficulties in making genetic predictions should apply to GEBVs in various environments. For accurate predictions, predictive models must consider terms that interact with various environments and environment x genotype interactions. These changed prediction models are divided into two types. The first is a non-informed model, which includes the environment as the main random effect. Also, the interaction between a genotype and environment is specified as a random effect. These prediction models report improved accuracy compared to traditional prediction models that specify only a genotype and environment as the main effects (Jarquin et al., 2014; Lopez-Cruz et al., 2015). On the other side, the informed prediction model includes measured environmental covariates in each environment. Then, informed prediction models incorporate information through variance-covariance structures using kernel-based methods. Finally, the informed prediction model calculates the interaction between each marker and environmental covariates and includes these calculations in predicting GEBVs. It is important to predict GEBVs by considering these models in various environments. Predictive models should be improved in terms of prediction results, or genetic and environmental variability should be analyzed to improve accuracy (Basnet et al., 2019). In general, traits with high heritability are determined by some genes having a major effect. Because these traits are less affected by the environment, their prediction accuracy is high. However, since numerous genes determine most of the traits humans try to breed with minor effects, it is necessary to consider the environment when selecting a predictive model (Combs and Bernardo, 2013).

#### 3.1.4 High-throughput phenotyping (HTP)

With the development of NGS and large-scale marker information, the application of GS increases in plants. However, one of the challenges that breeders face in training GS models is inaccurate phenotypic data. The sophistication of phenotypic information is as important as genomic information. Inaccurate phenotypic data make the predictive ability of the GS model decline. In addition, the existing phenotype measurement method has disadvantages because it is labor-intensive and requires a lot of time and costs. Recently, high-throughput phenotyping (HTP) has been used for accurate phenotypic information measurements. HTP is a non-destructive, fast, and accurate phenotypic measurement method that accurately captures traits of interest (Pabuayon et al., 2019). HTP has developed rapidly in plant breeding over the past decade. HTP processes stress tolerance, yield, and growth information through automated sensing, data acquisition, and processing. These advantages of HTP include accelerating the breeding cycle while allowing rapid screening of numerous plants at various growth stages (Yadav, 2021). The HTP platforms include RGB, normalized difference vegetation index (NDVI) sensors, multispectral and hyperspectral cameras, spectrometers, and light detection and ranging (LiDAR) technology (Yadav, 2021). The HTP platforms include RGB, normalized difference vegetation index (NDVI) sensors, multispectral and hyperspectral cameras, spectrometers, and light detection and ranging (LiDAR) technology (Shabannejad et al., 2020). Many studies on GS in plants using HTP have been conducted. GS was performed utilizing HTP using NDVI in wheat. Data collected through HTP showed a 7 to 33% increase compared to the standard univariate model (Crain et al., 2018). In addition, when HTP was performed in wheat using RGB, the prediction accuracy for days to maturity increased by 3 to 4 times (Shabannejad et al., 2020). Also, in another study, the measurement of secondary traits in wheat using an unmanned aerial vehicle (UAV) remote sensing increased the genetic prediction accuracy of grain yield by an average of 146% (Sun et al., 2019). These results suggest that using HTP improves model performance while enabling accurate selection. The efficient use of HTP becomes the background for ML and DL technology. The advanced combination of genotyping and phenotyping will provide breeders with opportunities for better GS.

### 3.2 Application of ML for GS

ML uses statistical techniques to allow systems to learn from data without explicitly programming them. ML takes a sample and then builds a model to explore algorithms that learn from current data and make predictions on new data. ML-based methods can be effective in improving prediction accuracy compared to conventional GS (Yoosefzadeh-Najafabadi et al., 2022). The main difference between the traditional statistics model and ML is that ML is a non-parametric model that offers tremendous flexibility to adapt to complex associations between data and outputs. It is difficult to build informative and predictive models because of the expanding scale of genome data, inherent complexity, the unique characteristics of organisms, and various environmental variables. Accordingly, the use of ML continues to grow and can be a good alternative (Greener et al., 2022). It initially adapts hidden patterns of unknown structures that cannot be incorporated into parametric models (Gianola, 2013).

GS statistical methods use ML for more accurate predictions (**Table 2**). Statistical analysis of the genetic basis of quantitative traits in plants is unsuitable for complex configurations such as pleiotropic genes, gene X gene, and gene X environment interactions. It is difficult to capture all marker effects, and problems such as the ‘large p, small n’ problem, sometimes lead to over-parameterization. ML methods improve prediction accuracy through observations of repeated experiences (Gianola et al., 2006). ML can identify hidden information in large data. Therefore, it is attractive for complex genomic information, including information about gene interactions and pleiotropic genes, when performing GS. ML develops and applies data through computer algorithms and is divided into supervised and unsupervised learning. Supervised learning predicts desired trait values from input data. On the other hand, unsupervised learning checks the group and association between input variables in which output variables do not exist.

**Table 2:** Machine learning application to GS in plants.

	Crop	Topology	Traits	Ref
1	Wheat	ANN	Grain development and hence morphometry	(Dubey et al., 2006)
2	Wheat & Pampas	ANN	Grain yield	(Alvarez, 2009)
3	Maize	ANN	Maize transpiration	(Fan et al., 2021)
		SVM		
4	Maize	ANN	Producing provinces	(Adisa et al., 2019)
5	Pepper	ANN	Fruit yield	(Gholipoor and Nadali, 2019)
6	Rice	ANN	Identification of 13 Rice Cultivars	(Abbaspour-Gilandeh et al., 2020)
7	Wheat	ANN	Quality parameters	(Mutlu et al., 2011)
8	Tomato	ANN	Escherichia coli	(Wang et al., 2008)
9	Wheat	SVM	Growth period	(Shen et al., 2021)
10	Wheat	SVM	Fusarium head blight	(Huang et al., 2019)
11	Wheat	SVM	Powdery mildew	(Huang et al., 2019)
12	Rice	SVM	Leaf diseases	(Sethy et al., 2020)
13	Maize	SVM	Root complexity	(Zhong et al., 2009)
14	Maize	SVM	Corn tassels	(Kurtulmus and Kavdir, 2014)
15	Tomato	SVM	Leaf Viruses	(Mokhtar et al., 2015)
16	Wheat	RF	Grain yield	(Roell et al., 2020)
17	Maize	RF	Grain yield	(Ramos et al., 2020)
18	Maize	RF	Total Nitrogen Content	(Lopez-Calderon et al., 2020)
19	Wheat & Rye	RF	5 leaf rust disease symptoms	(Wojtowicz et al., 2021)
20	Sweet pepper	RF	Fruit flavor	(Eggink et al., 2012)
21	Soybean	RBF, SVR, RF	Yield related components	(Yoosefzadeh-Najafabadi et al., 2022)

ANN, artificial neural network; SVM, support vector machine; RF, random forest; RBF, Radial basis function; SVR, Support vector regression; RF, Random forest.

Most ML involves supervised learning, such as Bayes nets, rule-based learning decision trees, naive Bayes, and nearest-neighbors, and applies to GS in the form of RF, ANN, and SVM (Gonzalez-Camacho et al., 2018). RF is an attractive alternative to analyzing complex individual traits using dense genetic markers. RF has good predictive power to measure the importance of each marker. RF does not require the specification of inheritance and takes into account non-additive effects. In addition, it is fast even when dealing with many covariates and can be applied to regression and classification models (Gonzalez-Recio et al., 2010).

Another ML-based model, SVM, is advantageous for classification and regression, similar to other ML models. The difference from other ML models is that SVM is specialized in identifying subtle patterns in complex and large amounts of information data. SVM makes a decision boundary with various feature vectors to achieve predictions. SVM can flexibly handle data using kernel-based functions. Furthermore, SVM can improve the non-linear form of phenotypes and genotypes using kernel functions (Noble, 2006). ML models can be applied in many scientific fields, but it is not clear whether these methods are superior to other statistical models. Therefore, if you want to make a clear prediction in the GS process, data acquired by various methods should be accumulated, and the optimal method should be selected based on experience.

Recently, the ML method, a specific type of ANN, has been considered to increase the performance of GS. ANN takes into account patterns in data and makes predictions about complex functions as universal approximations (Gianola et al., 2011). In GS, these functions automatically identify factors such as epistasis or dominance in genomic marker information. Moreover, it does not require any assumptions about the phenotypic distribution, and applying ANN to GS enables effective estimations of the effects of complex interactions (Rosado et al., 2020)

### 3.3 Application of DL for GS

Although studies on GS applied with DL are still lacking, several advanced studies exist (**Table 4**). In 2018, Montesinos-López et al. (Montesinos-Lopez et al., 2018). compared DL and GBLUP models using densely connected network architecture. Their study used nine published genomic data sets (three maize and six wheat data sets). When genotype x environment interactions (G x E) were ignored, DL had good predictive accuracy in 6 of 9 data sets. However, the prediction accuracy of GBLUP was excellent in 8 of 9 data sets when the G x E interaction was taken into account. In the study conducted in 2019, genomic-based prediction performance was confirmed by comparing the Bayesian threshold genomic best linear unbiased prediction (TGBLUP) model with multi-layer perceptron (MLP) and SVM methods (Montesinos-Lopez et al., 2019). It was confirmed that SVM and MLP were the most efficient in terms of computation time. These studies suggest that DL is not a data science panacea but a worthwhile addition to the data science toolkit in plant breeding. So far, research on GS using DL confirming prediction accuracy through the comparison with existing statistical models is limited. Thus, more research is needed.

An artificial neural network (ANN), the most fundamental concept in DL, is a network structure created by mimicking the neuron connection structure of a human neural network. A structure in which three or more ANNs are superimposed is called a deep neural network (DNN), and ML using this is called DL (LeCun et al., 2015). Popular DL topologies in GS include MLP, a convolutional neural network (CNN), and a recurrent neural network (RNN) (Montesinos-Lopez et al., 2021).

**Figure 4:**
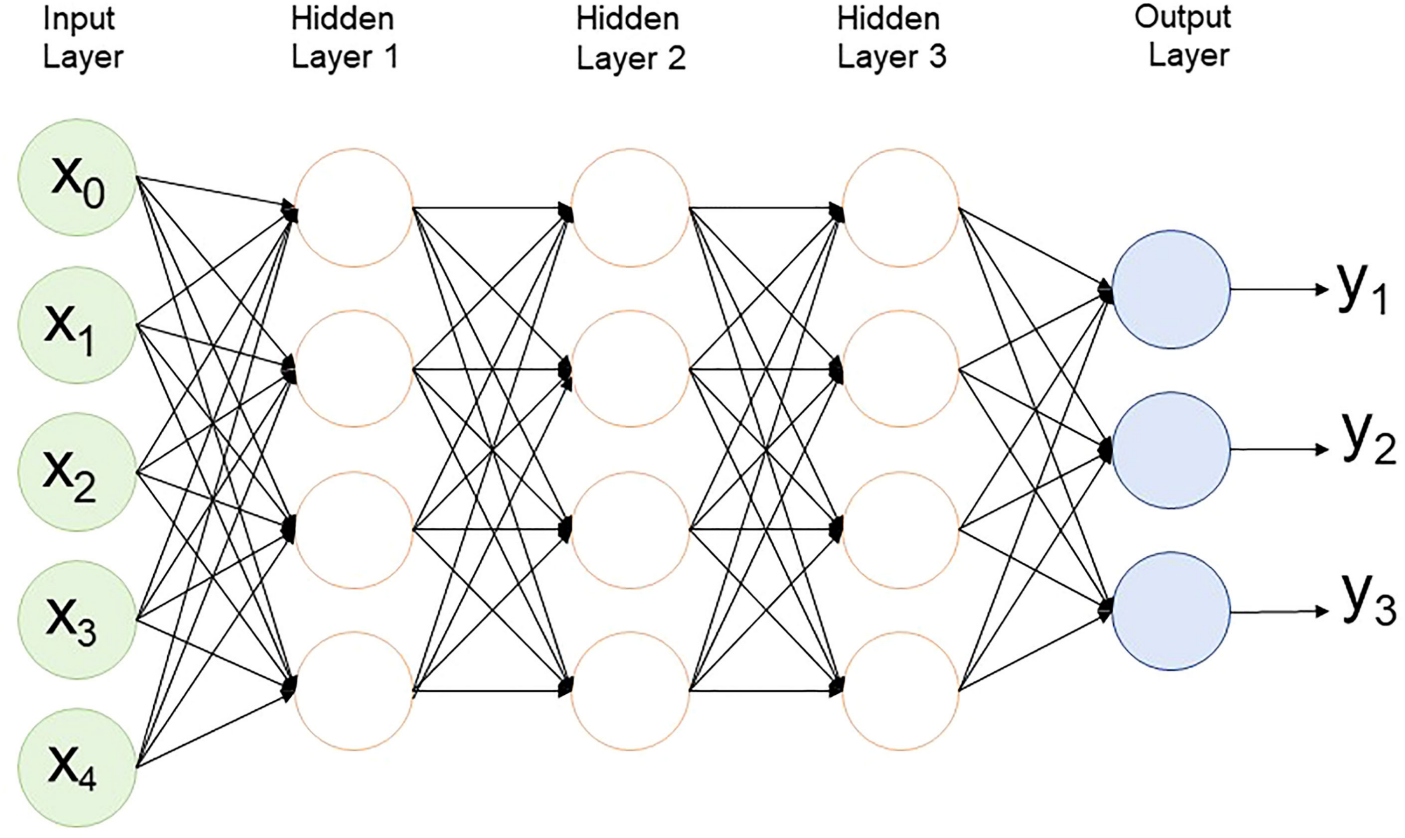
Multilayer perceptron structure with 3 hidden layers.

In MLP, in an artificial neural network, data moves in one direction from the input node through the hidden node to the output node (**Figure 4**). It has at least one hidden layer and is usually supervised learning. This method is the simplest to train, generally performs well in a variety of applications, and is suitable for general prediction problems where it is assumed that there is no special relationship between the inputs. However, these networks are prone to overfitting during the training process, so there is a problem in that the accuracy decreases in real data (Abdollahi-Arpanahi et al., 2020).

**Figure 5:**
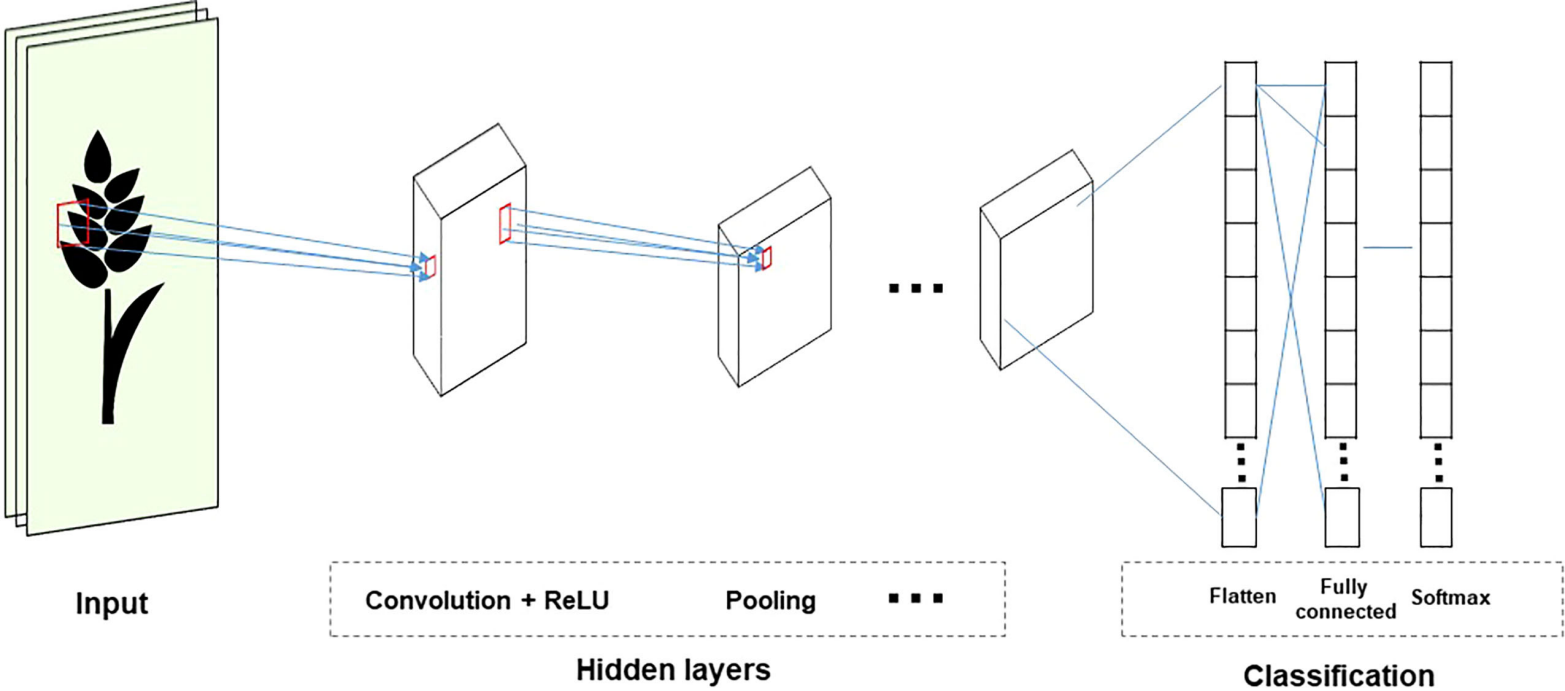
The structure of the CNN topology.

CNNs are used in visual recognition tasks involving images or video data. CNNs reduce the size of input and parameter sharing because the inputs are only partially connected to some neurons. Therefore, it is efficient because it reduces the parameters that need to be estimated. Most CNNs include three operations: convolution, non-linear transformation, and pooling (**Figure 5**). This process allows you to reduce the input size without losing relevant information. In addition, the training time can be decreased by reducing the parameters (Pook et al., 2020).

RNNs do not always travel in one direction, as they can be fed back to previous layers *via* synaptic connections. At least one feedback loop exists because the signal travels in both directions. Although the training parameters are reduced by sharing parameters across multiple steps, the short-term memory or latency of the network improves the performance, so training requires a lot of computational resources (Montesinos-Lopez et al., 2021).

Although few GS programs use DL, DL is emerging as a promising tool. First, the reason is that the DL model efficiently processes the image’s raw data without any preprocessing. Second, it captures naturally genetic diversity without specifying additional terms for the predictors. This is important for non-additive effects or complex relationships and interactions that are important to capture the genetic merit of the whole. Third, topologies such as CNNs efficiently capture the LD of neighboring SNPs. Fourth, some topologies, such as CNNs, share parameters so that more parameters do not need to be estimated, reducing the number of parameters that need to be estimated. However, there are a few caveats to using DL in GS. It is more prone to overfitting than existing statistical models. Research results have reported that these problems can be solved with a Bayesian paradigm (Neal, 1996). In addition, considerable knowledge is required to implement and output the DL model because it depends on the choice of many hyper-parameters and requires a very complex adjustment process.

The possibility that DLs can provide good predictive performance in GS has been suggested by several studies (**Table 3**). However, it still shows a similar or lower level of predictive performance than the existing statistical models. More iterative and collaborative experiments are needed, and more data are needed to utilize DL in genome selection. In addition, the data should include not only the phenotype but also various types of omics data, climate data, and experience data of breeders. Then we need to design an efficient topology for the DL model (Montesinos-Lopez et al., 2021).

**Table 3:** Deep learning application to GS in plants.

	Crop	Topology	Traits		Ref
1	Wheat	MLP	Grain yield, days to heading	(Perez-Rodriguez et al., 2012)	
2	Maize	DBN	Grain yield, female flowering or days to silking, male flowering time or days to anthesis, and anthesis-silking interval	(Gonzalez-Camacho et al., 2012)	
3	Maize and wheat	MLP	Grain yield	(Gonzalez-Camacho et al., 2016)	
4	Maize and wheat	MLP	Grain yield	(Montesinos-Lopez et al., 2018)	
5	wheat	MLP	Grain yield, days to heading, plant height	(Montesinos-Lopez et al., 2019)	
6	Maize	MLP	Grain yield, check yield, yield difference	(Wang, 2019)	
7	Soybean	CNN	Grain yield, protein, oil, moisture, plant height	(Liu et al., 2019)	
8	Arabidopsis	MLP, CNN	Arabidopsis traits	(Pook et al., 2020)	
9	Maize and wheat	MLP	Leaf spot diseases, Gray Leaf Spot	(Perez-Rodriguez et al., 2020)	

MLP, Multi-Layer Perceptron; CNN, Convolutional Neural Networks; DBN, Deep Belief Network.

## 4 Conclusion

Plant breeding has steadily increased crop productivity by developing superior varieties to support a growing population. Due to recent global environmental changes such as global warming, resource depletion, outbreaks of pests and diseases, and diversification of consumer demands, the role of plant breeding attracts much attention. Traditional breeding has developed dramatically and contributed to increased crop production, yield, and improved nutrition. Traditional breeding has created modern cultivars since the 20th century and has achieved great success in productivity. However, it is insufficient to meet the demand for crop production accompanied by exponential population growth. Breeding methods based on the traditional phenotypic selection are ineffective for low heritability and multi-genic quantitative traits (biological and abiotic stress, yield, and quality) because these traits are greatly influenced by the interaction between genes and the environment. Moreover, traditional breeding methods are time-consuming, laborious, and ineffective for cost and land use. In addition, low reliability and accuracy make these breeding methods less efficient. Accordingly, a new breeding method is required to quickly and accurately breed crops with high yield, good quality, and climate resilience. Researchers successfully established biotechnology-based molecular breeding technology to overcome the limitations of traditional breeding technology. They are now putting genome breeding technology into practice, also called predictive breeding. In summary, the history of plant breeding developed from traditional to molecular breeding. Predictive breeding will be available in the near future.

Rapid development in advanced technologies such as biotechnology, genomics, and phenomics improves progress in plant breeding. Breeders in the 21st century create mutations that directly correct target genes and expand the limits of genetic resources indefinitely by transcending the category of sexual reproduction with transformation technology. Selection in a population or lineage can rapidly and accurately fix the desired genotype using genomic information.

Integrating novel digital tools will be valuable and helpful in enhancing breeding progress, particularly for difficult-to-breed, quantitative traits. Vast genetic, genomic, phenotypic, and environmental data must be integrated and handled based on digital technology to fulfill the breeding technologies. In the current article, we first defined the term digital breeding, including breeding technologies from molecular to predictive breeding. For digital-based breeding technology to be commercialized, gene and genome information related to traits for each crop must be identified. It is expected that digital breeding can make it possible to reflect complex climates, geography, quantitative traits, and multiple traits in the breeding process. In a broad sense, therefore, digital breeding is not a single but a convergence technology. It can be defined as various attempts to actively utilize advanced technologies such as big data and artificial intelligence for agricultural breeding. However, as common confusion with new concepts in the early days of establishment, each researcher may have different ideas about digital breeding depending on their research field, research experience, and breeding resources. Particularly, some researchers think that DL-based techniques in breeding can become a game changer based on the results that DL has shown in various fields so far, so only DL-based techniques may be considered digital breeding. The different perceptions of digital breeding by researchers may cause confusion in the planning and promotion of related R&D. To address the problem, we first listened to the opinions of many researchers working with various breeding technologies. Consequently, we could classify the breeding level based on the intensity of digitalization. As a solution, we suggest that if digital breeding is divided like the autonomous driving levels in automobiles, it will be possible to easily organize the differences in thinking about digital breeding. Accordingly, we divided digital breeding into six phases. Phase 0 is traditional breeding that does not use digital technology and includes cross-breeding and mutation breeding. Phase 1 refers to identifying and processing substantial marker data generated by the development of genome sequencing technology. In Phase 2, markers are developed using bioinformatics tools. It is advantageous to select markers associated with qualitative traits using GWAS. Marker selection using GWAS has been actively carried out, but it is difficult to apply it to breeding directly. On the other hand, Phase 3 shows the potential to be practically applied to breeding. In this phase, the basis for practical application to breeding was laid by calculating the GEBVs using genomic information. Research based on GS started digital breeding. Although GS research has been actively conducted on livestock, it is still lacking in plants. Predictive statistical models for GS have been used in BLUP, LASSO, Bayesian, and ML-based models have also been applied. In Phase 4, breeding mainly uses DL to predict the phenotype by considering factors that affect plants, such as the environment. Some studies have been carried out by applying DL to plants, but it is still incomplete due to accuracy and technical problems. It looks like digital breeding, in the narrow sense, refers to this phase. In Phase 5, all processes from breeding design to phenotype prediction are automatically performed using DL. It has not yet been studied and is the ultimate goal for digital breeding techniques to evolve (**Table 4**). More phases can be added if new technologies emerge in the future.

**Table 4:** Phases of digital breeding defined in the current review.

Phase	Definition	Associated Statistical Model	Associated Breeding Techniques
0	No digital technology used	–	Traditional breeding
1	Digitize large amounts of data for marker development	–	Molecular breeding
2	Limited use of digital technology to develop markers	GWAS (GLM, MLM, FarmCPU, etc.)	Molecular breeding
3	Prediction of GEBVs mainly based on quantitative traits	GS (BLUP, LASSO, Bayesian, Machine learning, etc.)	GS
4	Prediction of phenotype considering environments	Phenotype prediction (ML, DL)	GS
5	Using artificial intelligence technology from breeding design to phenotype prediction	Automated breeding design of all processes (DL)	AI breeding

## Author contributions

CK, T-HL, and DJ designed and structured the review, DJ, YK, SL, SC, and YS collected the information, organized the figures and tables, and wrote the original draft. CK and T-HL reviewed the manuscript. DJ edited and wrote the manuscript. All authors contributed to the article and approved the submitted version.
